# Supporting the Identification of Novel Fragment-Based Positive Allosteric Modulators Using a Supervised Molecular Dynamics Approach: A Retrospective Analysis Considering the Human A2A Adenosine Receptor as a Key Example

**DOI:** 10.3390/molecules22050818

**Published:** 2017-05-16

**Authors:** Giuseppe Deganutti, Stefano Moro

**Affiliations:** Molecular Modeling Section (MMS), Department of Pharmaceutical and Pharmacological Sciences, University of Padova, Via Marzolo 5, 35131 Padua, Italy; giuseppe.deganutti@phd.unipd.it

**Keywords:** adenosine receptors, positive allosteric modulators, fragment-based approaches, supervised molecular dynamics

## Abstract

Structure-driven fragment-based (SDFB) approaches have provided efficient methods for the identification of novel drug candidates. This strategy has been largely applied in discovering several pharmacological ligand classes, including enzyme inhibitors, receptor antagonists and, more recently, also allosteric (positive and negative) modulators. Recently, Siegal and collaborators reported an interesting study, performed on a detergent-solubilized StaR adenosine A_2A_ receptor, describing the existence of both fragment-like negative allosteric modulators (NAMs), and fragment-like positive allosteric modulators (PAMs). From this retrospective study, our results suggest that Supervised Molecular Dynamics (SuMD) simulations can support, on a reasonable time scale, the identification of fragment-like PAMs following their receptor recognition pathways and characterizing the possible allosteric binding sites.

## 1. Introduction

Structure-driven fragment-based (SDFB) approaches have provided efficient methods for the design of novel pharmacological probes as well as drug candidates [1]. One of the main advances of using fragment-based approaches is that they rely on screening small chemical fragments (100–250 Da) [2] allowing one to explore a significantly larger portion of chemical space with fewer compounds when compared with other screening strategies [2]. Moreover, early hits from a fragment screen generally bind more efficiently, even if not strongly, to their target and represent desirable starting points for medicinal chemists to grow and optimize these into lead candidates.

Interestingly, this strategy has been largely applied in discovering several classes of pharmacological ligands, including enzyme inhibitors, receptor antagonists and, more recently, also allosteric (positive and negative) modulators [2,3,4,5]. In particular, allosteric modulation of G protein-coupled receptors (GPCRs) has stimulated an intensive campaign to identify new classes of hit-candidates different from conventional agonists and antagonists, in particular considering the breakthroughs coming from GPCR crystallographic determination that has resulted in a fast-growing number of structures obtained as complexes with allosteric modulators. It is useful to underline the Allosteric Database (ASD) repository that it has been developed to provide comprehensive information characterizing allosteric regulation ranging from allosteric proteins, modulators of interactions, sites, pathways, functions and related diseases [6,7]. Thus, elucidating the profile of allosteric recognition in GPCRs provides hope for the design of potent modulators with improved protein subtype selectivity, adverse effects, and/or pathway-biased signaling. This has been the subject of several recent reviews [8,9].

Recently, Chen and collaborators reported an interesting study in which a screen of 531 fragments was performed on a detergent-solubilized StaR adenosine A_2A_ receptor [10]. Several hits, with both orthosteric and allosteric modulatory activity, were successfully identified and then thoroughly characterized for biochemical activity using the wild-type receptor. The authors reported that three fragments significantly (at least 30%) increased the *k*_off_ of the orthosteric ligand and hence are negative allosteric modulators (NAMs), while four significantly decreased the *k*_off_ and hence are positive allosteric modulators (PAMs) of both ZM241385 and NECA [10].

We have recently reported on an alternative computational method, Supervised Molecular Dynamics (SuMD), that allows investigating the ligand-receptor recognition pathway on a nanoseconds (ns) time scale [11,12,13]. SuMD performs short unbiased MD simulations during which the distance between the center of masses of the ligand atoms and the binding site is monitored. At the end of each time window distance points collected are fitted into a linear function f(x) = *m*x and a tabu-like algorithm is applied to increase the probability to produce ligand-receptor binding events as follows: if the slope (*m*) is negative, the ligand is likely to move closer to the binding site and a classic MD simulation is restarted from the last set of coordinates and velocities. Otherwise, the simulation is restored from the original set of coordinates and random velocities are reassigned to each atom. The supervision is repeated until the ligand-receptor distance is less than 5 Å. In addition, to speeding up the acquisition of the ligand–receptor recognition trajectory, this approach facilitates the identification and the structural characterization of multiple binding events (such as *meta*-binding, allosteric, and orthosteric sites) by taking advantage of the all-atom MD simulations accuracy of GPCR–ligand complexes embedded into explicit lipid–water environment. In particular, we have utilized SuMD with the aim to characterize and rationalize the activity of the LUF6000, an adenosine A_3_ receptor PAM, at a molecular level [14]. We have analyzed the ligand–receptor recognition pattern, both for LUF6000 and the endogenous agonist adenosine separately and also considering the recognition pathway of the PAM by the hA_3_ AR in complex with adenosine. In this work, to verify the applicability domain of our methodology, we selected two fragment-like adenosine A_2A_ receptor PAMs (see Table 1) exploring their possible recognition pathways by performing SuMD simulations in the absence and in presence of the NECA agonist. Interestingly, from this retrospective study, our results suggest that SuMD simulations can support, in a reasonable time scale, the identification of a fragment-like PAMs following their receptor recognition pathways and characterizing the possible allosteric binding sites.

## 2. Results

Here we employed supervised molecular dynamics (SuMD) [11,12], a computational technique that allows simulating intermolecular recognition events on a nanoseconds time scale, with the aim of rationalizing the experimental behavior of ZB1854 [3-(3,4-dihydroquinolin-1(2*H*)-yl)propanoic acid)] and ZB268 [(6-chloro-2*H*-chromene-3-carboxylic acid] (Table 1), two fragments (PM < 300 Da) able to act as A_2A_ AR PAM. In the experimental work by Chen et al. [10] the modulation exerted by these two fragments on both agonists NECA and CGS21680 as well on the antagonist ZM241385 highlighted their ability to slightly slow down the unbinding kinetics constants *k*_off_ of orthosteric ligands (Table 1).

In the present study, using the recently disclosed X-ray crystal structure of the A_2A_ AR subtype in its active G protein-bound conformation [15], we sampled multiple putative allosteric binding sites by means of SuMD simulations and then monitored the stability of the orthosteric complex between NECA and the A_2A_ AR (see Appendix A for its description) by using unsupervised MD. Considering that ZB1854 and ZB268 bring an acidic moiety, we decide to simulate the binding from the extracellular side of the simulation box. Indeed, at physiological pH values, the fragments prevalently exist in their anionic form, not allowing the passive molecular diffusion through biological membranes. We also performed a SuMD simulation for caffeine (which is a well-known fragment able to act as an antagonist on A_2A_ AR [16]) and ZB1854 on the apo form of A_2A_ AR, with the aim of understand why experiments excluded any antagonist activity for these fragments.

### 2.1. SuMD Simulations of ZB1854 on the APO Form of A_2A_ AR

**Figure 1:**
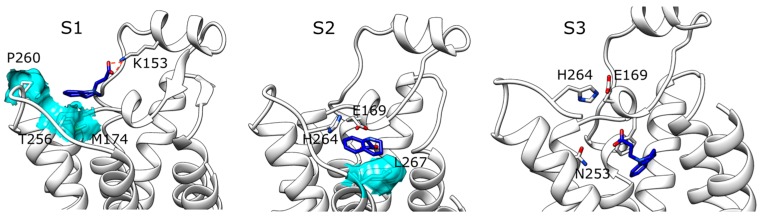
ZB1854-A_2A_ AR (apo form) recognition during SuMD replica 2. (**S1**) Meta-stable complex; (**S2**) intermediate energetically unfavorable state; (**S3**) ZB1854 inside the A_2A_ AR orthosteric site (TM6 has been removed for clarity).

During SuMD simulations performed on compound ZB1854 (Video S2), in all of the five replicates the fragment approached an extracellular receptor spot, located at about 15 Å from the orthosteric site and edged by EL2, EL3, TM5 and TM6 (Figure S1). SuMD replicas 1 (Figure S2 panel A) and 3 (Figure S2 panel B) terminated with the ligand still in this extracellular region, while during replica 2, 4 and 5 it was able to partially reach the orthosteric site. The high energetic stabilizations gained in these stable sites are mainly ascribable to ionic interactions (notably, it is well known that molecular mechanics models overestimate this kind of intermolecular interactions [17]) between the acidic moiety of the PAM and the positively charged residues K153 (EL2) and H264 (EL3). Interestingly, three out of five SuMD simulations resulted in an orthosteric binding event. However, considering the recognition events energy landscapes (Figure S2) it is important to note both that the fragment usually did not reach a deep position in the binding site (a few points are below the distances of 5 Å) and that the metastable states were usually more stable than the orthosteric site. Moreover, during the trajectories some distances between the metastable and the orthosteric site were poorly populated, indicating the presence of energetically unfavorable configurations (ascribable to putative binding transition states). SuMD replica 2 (Figure S1 panel B) well represents this scenario. The recognition pathway can be summarized in three main steps: (i) ZB1854 experienced a stabilization in a meta-stable state S1 (Figure 1 panel S1), establishing an electrostatic interaction with K153 (EL2) and hydrophobic contacts with M174, P260 and T256; (ii) the fragment experienced the energetically unfavourable state S2 (Figure 1 panel S2) characterized by an electrostatic repulsion with E169 (the only stabilizing contact is formed with L267); (iii) finally, the fragment reached the orthosteric site in conformation S3 (Figure 1 panel S3), engaging F168 in a hydrophobic contact. Notably, the compound did not interact with N253, which is one of the A_2A_ AR residues necessary for binding of both agonists and antagonist [18,19]. Among all the five SuMD replicates, the last one (Figure S1 panel E) is the only one that ended with a ZB1854-A_2A_ AR complex more stable than the metastable states along the recognition pathway. This stabilization was due to an electrostatic interaction with the positively charged H264 side chain, but again no fundamental interactions were established with N253 (Figure S3). It is possible to highlight unfavorable states (located at about 10 Å from the orthosteric site) also during SuMD simulations for caffeine (Figures S4 and S5). However, caffeine in its final bound state is usually more stable than in the former intermediate states and it is able to reach deep positions in the orthosteric site at the end of the simulations (many points below the distances of 5 Å in Figure S4). All these differences should allow distinguishing a well-known A_2A_ AR binder (caffeine) from a compound that is not able to form stable orthosteric complexes (ZB1854).

### 2.2. SuMD Simulations of ZB1854 on A_2A_ AR in Orthosteric Complex with NECA

**Figure 2:**
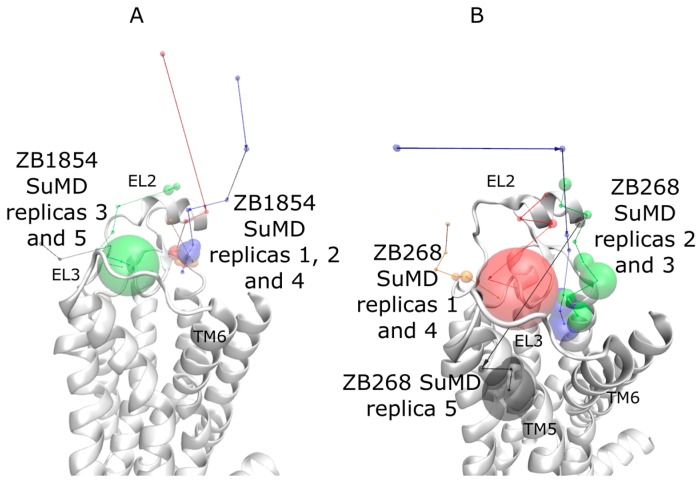
Recognition pathways, according to five SuMD replicas for (**A**) ZB1854-A_2A_ AR; (**B**) ZB268-A_2A_ AR. Segments approximate the ligands’ trajectories, while the diameters of the spheres are proportional to the SuMD simulation time spent by the ligand in the corresponding position. Replica 1 is red color coded; replica 2 is blue color coded; replica 3 is green color coded; replica 4 is orange color coded; replica 5 is black color coded.

**Figure 3:**
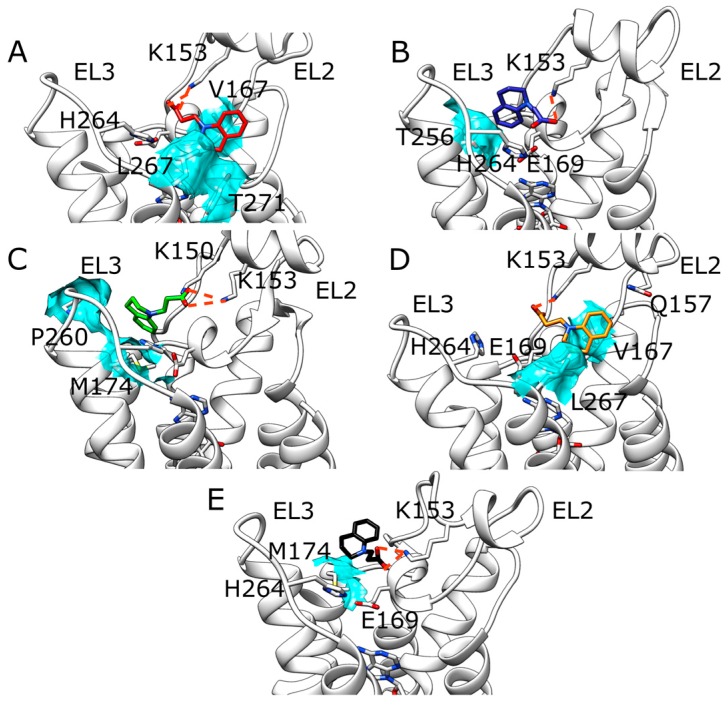
ZB1854 five putative allosteric sites detected during SuMD simulations (NECA is present in the orthosteric site). Polar interactions are highlighted as dotted lines, while the main hydrophobic contacts are depicted as cyan transparent surfaces. (**A**) Replica 1; (**B**) replica 2; (**C**) replica 3; (**D**) replica 4; (**E**) replica 5.

Fragment ZB1854 showed a concentration-dependent modulatory effect on the dissociation of [^3^H]-NECA from the A_2A_ AR (Table 1) and potentiated the maximal effect of CGS21680 [10]. During five SuMD replicates (Video S3 and Figure S6) ZB1854 approached the receptor extracellular vestibula in two main positions (Figure 2 panel A), located at about 15 Å from the orthosteric site. More precisely, in replicas 1, 2 and 4 it reached a site located between EL2 and TM7, while in replicas 3 and 5 the PAM arrived at a spot enclosed by EL2 and EL3, in analogy with SuMD simulations on the apo form. Unsupervised MD simulations performed on each final complex did not highlight any evident further orthosteric stabilization between the NECA and the A_2A_ AR (Figure S7). Indeed, it is difficult to distinguish normal dynamic fluctuations of the intermolecular interactions from stabilizations/destabilizations that could be caused by the binding of the fragment to extracellular elements of the receptor. The five putative allosteric sites sampled for ZB1854 are better shown in Figure 3. As anticipated, the interaction between the ligand and K153 side chain are present in all the five intermolecular complexes. What differentiates these configuration is the position of the dihydroquinoline scaffold: in replica 2 it is oriented toward T256 (Figure 3, panel B), in replica 3 it makes contacts with M174 and P260 (Figure 3, panel C), in replica 5 interacts with M174 side chain (Figure 3, panel E) and in replicas 1 and 4 it is stacked between V167 and L267 side chains (Figure 3, panels A and D). Interestingly, the latter configuration partially overlaps with the accessory binding site observed in a recent A_2A_ AR X-ray crystal structure [20], which comprises L267 and Y271 side chains.

### 2.3. SuMD Simulations of ZB268 on A_2A_ AR in Orthosteric Complex with NECA

**Figure 4:**
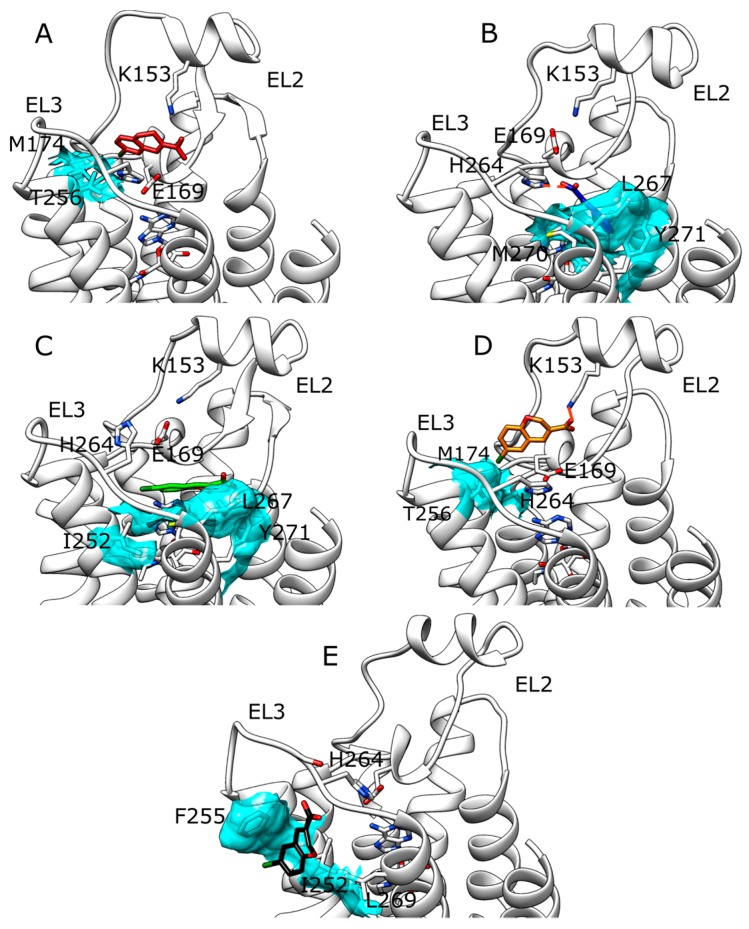
Five putative allosteric sites detected at the end of SuMD simulations for ZB268 (NECA is present in the orthosteric site). Polar interactions are highlighted as dotted lines, while the main hydrophobic contacts are depicted as cyan transparent surfaces. (**A**) Replica 1; (**B**) replica 2; (**C**) replica 3; (**D**) replica 4; (**E**) replica 5.

Compound ZB268 showed a concentration-dependent modulatory effect on the dissociation of [^3^H]-NECA from the A_2A_ AR slightly lower than ZB1854 (Table 1) [10]. During five SuMD replicas, ZB268 reached the extracellular vestibula and stopped at about 10–15 Å (Figure 1 panel B), establishing stable complexes with A_2A_ AR (Figure S8 panels A–E). Replica 5 (Figure S8 panel E) resulted in the less stabilized intermolecular complex (described by force field interactions in the range of 0–45 kcal/mol), due to the fact that the fragment reached the receptor surface outside the TMs bundle (Figure 4 panel E). During unsupervised MD simulations run on these configurations, no evident further orthosteric stabilization between NECA and the receptor was observed (Figure S9 panels A–E). SuMD replica 1 (Figure 4 panel A) and replica 4 (Figure 4 panel D) led to the formation of a similar complex, characterized by the hydrophobic contacts with T256 and M174 as well as ionic interaction with K153. Replica 2 (Figure 4 panel B) and replica 3 (Figure 4 panel C), instead, terminated with the PAM fragment able to reach a hydrophobic pocket edged by Y271, L267 and M270 side chains. However, while during replica 3 the ligand oriented its chlorine atom towards I252 and H264 side chains, in replica 2 the carboxylic moiety of ZB268 interacted with the protonated H264. As observed for compound ZB1854, this latter configuration well overlaps with the accessory binding site observed in the work of Sun et al. [20] which comprises L267 and Y271 side chains. SuMD replica 5 produced a peculiar complex between A_2A_ AR and ZB268 (Figure 4 panel E), in which the fragment made contacts with residues located towards the lipid environment, in correspondence of both TM6 (F255 and I252) and TM7 (L269). In a recent work, Segala et al. [21] addressed the role of the salt bridge formed by H264 (EL3) and E169 (EL2) in modulating the dissociation rates of different A_2A_ AR antagonists, correlating the ligands’ residence times to the stability of the salt bridge itself. Inspired by their approach we applied the same metadynamics protocol for an in silico evaluation of putative stabilizations produced by the binding of ZB268 at the protein EL3/lipid phase interface. Compared to the complex A_2A_ AR-NECA in absence of the PAM, simulations highlighted a slight stabilization of the salt bridge in its closed conformation when the PAM is present (a distance of about 4–5 Å), as reported in Figure S10.

## 3. Discussion

Nowadays increasing efforts are addressed to a deeper understanding of GPCRs allosterism. Results from a wide range of both experimental and theoretical studies (e.g., mutagenesis, X-ray crystallography, spectroscopy, bioinformatics, and modeling) have recently allowed depicting a more general picture of this phenomenon [6,7]. However, focusing on the A_2A_ AR, still few information have been reported about allosteric sites [22]. It is well accepted that sodium ions are able to unselectively stabilize class A GPCRs in inactive conformations [23,24], while drug design strategies led to the characterization of more selective compounds by using amiloride as reference compound able to bind the sodium allosteric site [25]. Indeed, as (to the best of our knowledge) alternative A_2A_ AR allosteric sites have not still been spotted or proposed, in this paper, we have computationally addressed this topic. Inspired by our work on the A_3_ AR PAM LUF6000 [14], by means of supervised molecular dynamics (SuMD) simulations, we obtained insights about putative allosteric sites for compounds ZB1854 and ZB268 on the A_2A_ AR subtype. Indeed, these fragments are able to affect the experimental NECA-A_2A_ AR dissociation kinetics *k*_off_ slowing down the orthosteric unbinding [10]. According to Eyring’s theory [26], the experimental *k*_off_ decrease observed in presence of these PAMs (18% and 8% respectively) is caused by a free energy increase of less than 1 kcal/mol between the NECA bound state and the transition state of the overall unbinding process. Notably, this restricted energetic alteration can be due either to a destabilization of the transition state (which requires more energy to be overcome) or a further stabilization of the bound state.

Outcomes from SuMD performed on A_2A_ AR apo form and ZB1854 confirms the experimental observation that the fragment does not exert any antagonism towards the receptor. The orthosteric binding should be kinetically unfavorable due to the presence of the acidic residue E169 on EL2, able to electrostatically repulse the negatively charged compound and therefore generating a high-energy transition state. Moreover, the hypothetic orthosteric complex sampled lacked the stabilization generated by hydrogen bonding with an N253 side chain, key residues for binding to A_2A_ AR [18,19]. Interestingly, while in absence of NECA ZB1854 approached the receptor preferentially at the top of TM4, TM5 (between EL3 and EL2), in presence of the orthosteric agonist the PAM experienced an alternative binding mode, partially corresponding to the accessory site observed in a recent A_2A_ AR X-ray crystal structure [20]. In both of the two proposed configurations, residue K153 played a crucial role stabilizing the complex through an ionic interaction. During SuMD simulations and unsupervised MD simulation, the stability of NECA-A_2A_ AR orthosteric complex was subjected to normal dynamic fluctuations, making difficult to observe potential slight contributions produced by fragments binding to the receptor. It is more reliable that the fragments should obstacle the dissociation of the ligand by weakly bind to sites located on the receptor extracellular surface (interestingly, PAM effect was observed on both agonists and antagonists). Indeed, ZB1854 and ZB268 recognitions energy landscapes (Figures S6 and S8) suggest an almost diffusive binding kinetics, as the fragments reached the receptor without any bottleneck along their pathways. Moreover, in a recent SuMD applicative study [12] different NECA-A_2A_ AR recognition pathways were sampled, highlighting metastable sites located at the top of TM5 and TM6, in positions consistent with the putative allosteric site detected during replica 3 and 5 (fragment ZB1854) as well as during replicas 1 and 4 (fragment ZB268). According to SuMD simulation replica 5, an alternative PAM mechanism for ZB268 was proposed. In this case, ZB268 binding to an external protein site at the lipid interface may generate a slight stabilization of the closed conformation of the H264 (EL3)-E169 (EL2) salt bridge, which contributes to the overall dissociation kinetics rates of A_2A_ AR ligands.

## 4. Materials and Methods

### 4.1. General

All computations were carried out on a hybrid CPU/GPU cluster. Molecular dynamics (MD) simulations were performed with ACEMD engine [27] on a GPU cluster composed by 20 NVIDIA drivers: five NVIDIA GTX 980Ti, seven NVIDIA GTX 980, three NVIDIA GTX 780, two NVIDIA GTX 680 and three NVIDIA GTX 580. For all the simulations, the CHARMM27 [28]/CHARMM general force field (CGenFF) combination was adopted [29,30].

### 4.2. Systems Preparation

NECA-A_2A_ AR complex and caffeine-A_2A_ AR were retrieved from the RCSB Protein Data Bank database [31] (PDB ID 5G53 [15] and 3RFM [16] respectively) and handled by means of the MOE [32] protein structure preparation tool. Hydrogen atoms were assigned according to Protonate-3D [33] and any missing loop was modeled with the homology modeling protocol. Missing atoms in the side chains, as well as non-natural N-terminal and C-terminal, were rebuilt according to the CHARMM force field topology [28]. In the case of PDB ID 5G53, the guanosine-5′-diphosphate (GDP) bound to the engineered G-protein was removed. A_2A_ AR apo forms were obtained by simply deleting NECA and the caffeine from their respective complex.

### 4.3. Ligand Parameterization

NECA, ZB1854, ZB268, ZB418 and caffeine force field parameters were initially retrieved from the Paramchem web service and then deeply optimized in concordance with CGenFF [30], at the MP2/6-31G* level of the theory [34] by using Gaussian 09 and RESP partial charges [35].

### 4.4. Solvated System Setup and Equilibration

Systems were embedded in a 1-palmitoyl-2oleyl-*sn*-glycerol-3-phospho-choline (POPC) lipid bilayer, according to the pre-orientation provided by the Orientations Proteins in Membrane (OPM) database [36] and by using the VMD [37] membrane builder plugin. Lipids within 0.6 Å from the protein were removed and TIP3P model [38] water molecules were added to solvate the system by means of Solvate1.0 [39]. System charge neutrality was reached by adding Na^ì^/Cl^−^ counterions to a final concentration of 0.154. Equilibration was performed through a three-step procedure. In the first one, 1500 conjugate-gradient minimization steps were applied in order to reduce the clashes between protein and lipids. Then, a 5 ns long MD simulation was performed in the NPT ensemble, with a positional constraint of 1 kcal mol^−1^ Å^−2^ on ligand, protein, and lipid phosphorus atoms. During the second stage, 20 ns of MD simulation in the NPT ensemble were performed constraining all the protein and ligand atoms but leaving POPC residues free to diffuse in the bilayer. In the last equilibration stage, positional constraints were applied only to the ligand and protein backbone alpha carbons for further 5 ns of MD simulation.

All the MD simulations were performed using: (1) an integration time step of 2 fs; (2) the Berendsen barostat [40] in order to maintain the system pressure at 1 atm; (3) the Langevin thermostat [41] to maintain temperature at 310 K with a low dumping of 1 ps^−1^; (4) the M-SHAKE algorithm [42] to constrain the bond lengths involving hydrogen atoms.

### 4.5. Supervised Molecular Dynamics (SuMD) Simulations

We performed supervised molecular dynamics (SuMD) simulations [11,12] in order to sample putative binding sites for allosteric modulators ZB1854, ZB268 and ZB418, as well to simulate the binding of caffeine to A_2A_ AR. According to this MD-based approach, the timescale needed to reproduce complete intermolecular complexes formations results in the range of nanoseconds, instead of hundreds of nanoseconds or microseconds usually necessary with unsupervised MD. Sampling is gained without the introduction of any bias, but just by applying a tabu—like algorithm to monitor the distance between the centers of masses of the ligand and the binding site, during short unbiased MD simulations. SuMD considers the ligand atoms and the atoms of user-defined protein residues to monitor the distance between the center of masses of the binder and the binding site. A series of 600 ps unbiased MD simulations are performed and after each simulation, the distance points collected at regular time intervals are fitted into a linear function. If the resulting slope is negative the next simulation step starts from the last set of coordinates and velocities produced, otherwise the simulation is restarted by randomly assigning the atomic velocities. Short simulations are perpetuated under the supervision until the distance between the ligand and the binding site drops below 5 Å, then the supervision is disabled and a classical MD simulation is performed. From a general point of view, SuMD can be considered an adaptive sampling method during which unbiased simulation are run consecutively, instead of that in parallel as usually done [43,44]. In the present study for the computation of the orthosteric center of mass, we considered A_2A_ AR residues N253, F168, W246, and T88.

### 4.6. Metadynamics Simulations

The stability of the salt bridge formed by H264 (EL3) and E169 (EL2) was investigated by means of metadynamics simulations [45,46] by using Gromacs 5.1.2 [47], PLUMED 2.3 [48] and CHARMM36 force field [29,49]. A_2A_ in complex with NECA and A_2A_ in complex with NECA and ZB268 (configuration resulted from SuMD replica 5) were subjected to the same computational protocol (consistent with the one from the work of Segala et al. [21]). Each system was embedded in a triclinic simulation box, consisting of an equilibrated membrane formed by POPC (1-palmitoyl-2-oleoyl-*sn*-glycero-3-phosphocholine) lipids and TIP3 water molecules, using g_membed [50]. Na^+^ Cl^−^ ions were added to neutralize the system. An energy minimization protocol consisting of 1000 steps steepest-descent algorithm has been applied to the system. The membrane was equilibrated using 0.5 ns MD simulation, restraining all bonds of protein and ligands by using LINCS algorithm [51]. The MD was performed in the NPT ensemble, maintaining the temperature at 298 K using v-rescale [52] and the pressure of 1.013 bars using the Parrinello-Rahman [53] approach. Without applying any positional restraints, the system has been equilibrated by gradually increasing the temperature from 29.8 K to 298K in 3 ns. During 10 ns of productive well-tempered [54] metadynamics simulations (temperature was kept at 298 K, the bias factor was set to 6 and the deposition frequency was 0.5 ps) two collective variables (CVs) were biased: (i) CV1 was the distance between C5 of H264 and Cδ of E169 and (ii) CV2 was H264 dihedral angle defined by Cα, Cβ, C4 and C5 atoms. The initial Gaussian energy bias was: (i) 0.3 kcal/mol height and 0.04 Å width in the case of CV1; and (ii) 0.3 kcal/mol height and 0.3 rad width in the case of CV2.

## Figures and Tables

**Table 1:** Fragments with PAM activity towards A_2A_ AR considered in the present study [10].

Compound	ID	[^3^H]-NECA *k*_off_ (min^−1^ or % Allosteric Modulation)
No PAM	/	0.015 ± 0.02
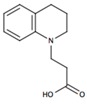	ZB1854	0.0095 ± 0.0005 (18%)
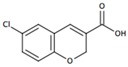	ZB268	8%

Data from [10].

**Figure A1:**
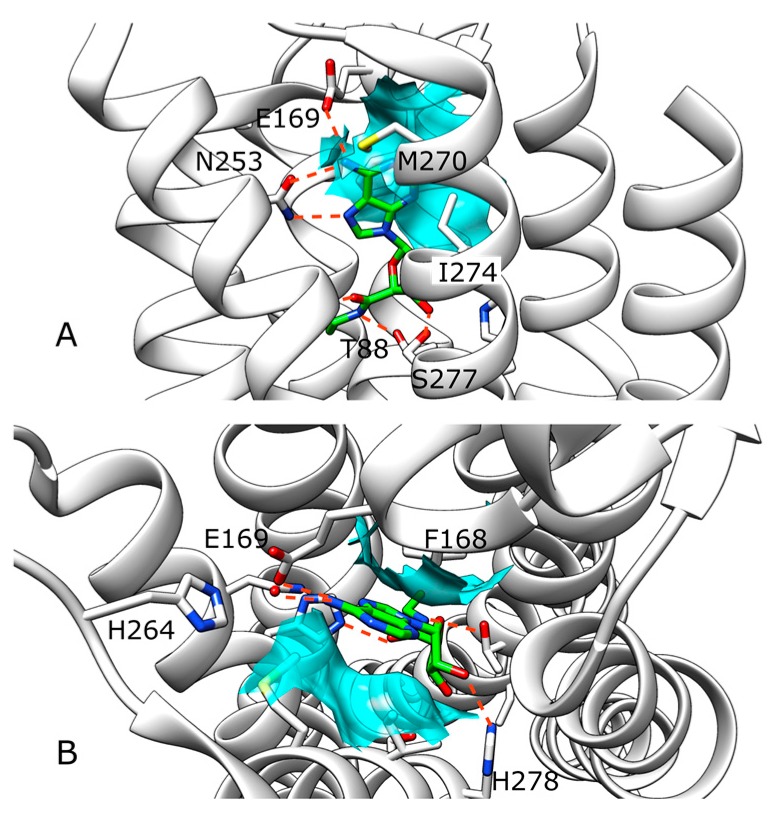
NECA-A_2A_ AR intermolecular complex. Polar interactions are highlighted as dotted lines, while the main hydrophobic contacts are depicted as cyan transparent surfaces. (**A**) Side view from the lipid bilayer; (**B**) extracellular side view.

## Supplementary Materials

Supplementary materials are available online.

## Appendix A

In its A_2A_ AR orthosteric complex, NECA is stabilized by several interactions (Figure A1). Hydrogen bonds are established between the purine scaffold and N253 and E169 side chains, while the ethylcarboxamidoribose moiety engages H250, H278, S277 and T88 side chains. Hydrophobic contacts are ascribable to F168, I274 and M270 side chains.
