# Supplementary material for: Supporting the Identification of Novel Fragment-Based Positive Allosteric Modulators Using a Supervised Molecular Dynamics Approach: A Retrospective Analysis Considering the Human A2A Adenosine Receptor as a Key Example
Source: Molecules. 2017 May 16;22(5):818. doi: 10.3390/molecules22050818 (PMC6154550; doi:10.3390/molecules22050818)
Supplement: Supplementary file 1 [file molecules-22-00818-s001.zip › molecules-186215 supplementary.pdf]

Supplementary Materials: Supporting the identification of novel fragment-based positive allosteric modulators using a supervised molecular dynamics approach: a retrospective analysis considering the human A2A adenosine receptor as a key study

Giuseppe Deganutti and Stefano Moro

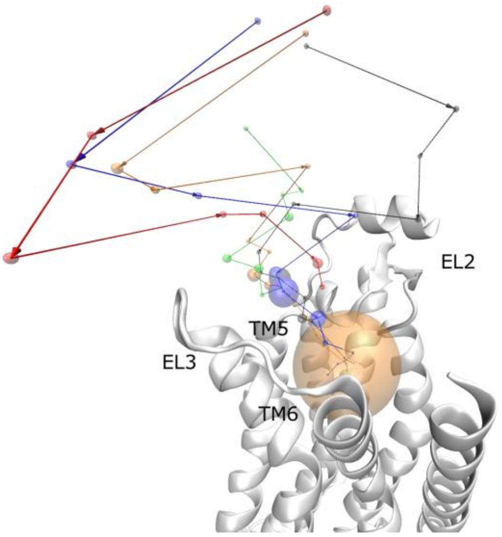

Figure S1. ZB1854 - A_{2A} AR (apo form) recognition pathways, obtained from five SuMD simulation replicas. Replica 1 is red color coded; replica 2 is blue color coded; replica 3 is green color coded; replica 4 is orange color coded; replica 5 is black color coded.

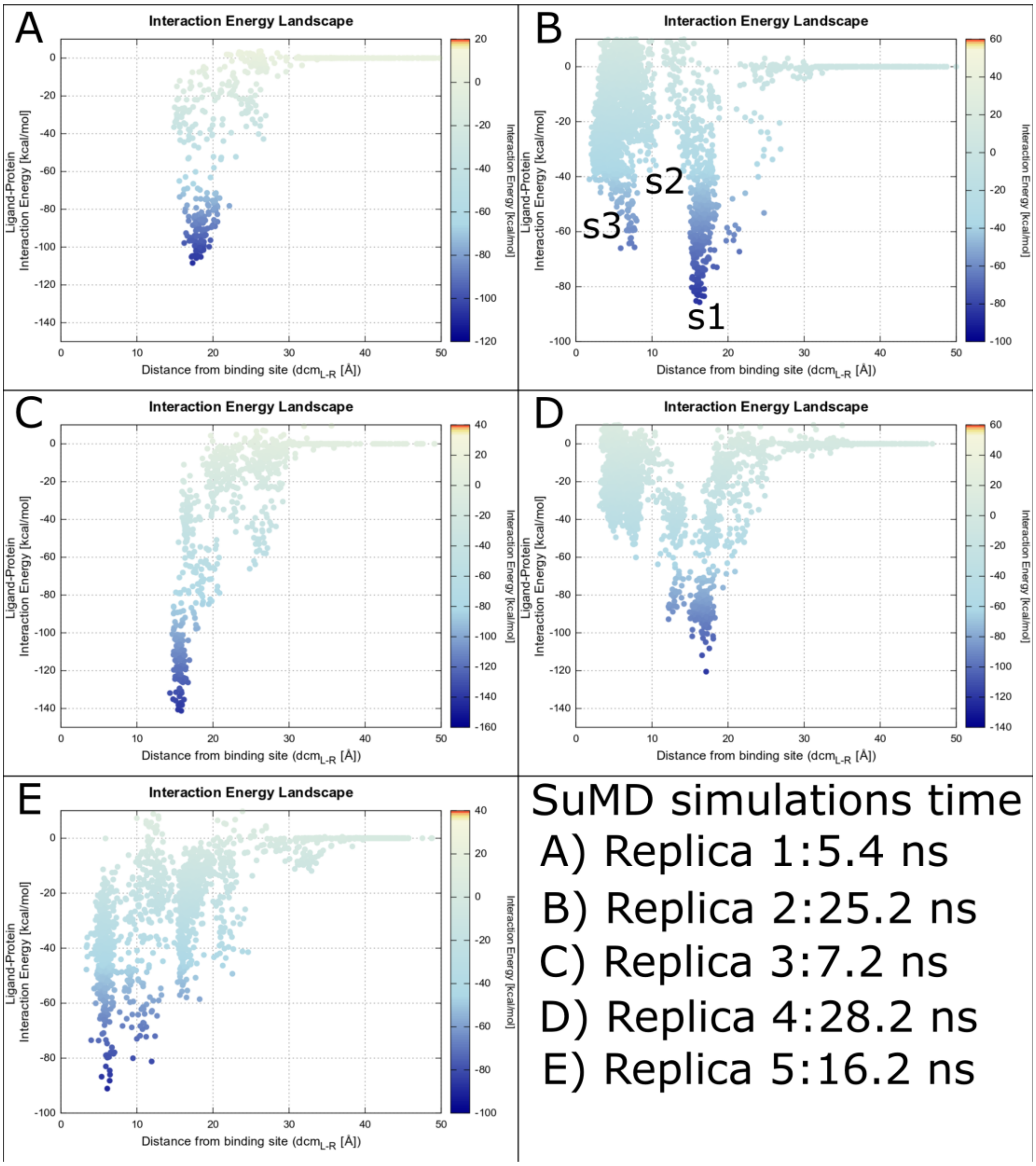

Figure S2. ZB1854 - A_{2A} AR (apo form) recognition energy landscapes for the five SuMD replicas performed. On the x axis is reported the ligand distance from the orthosteric site, while the y axis shows the intermolecular interaction extracted from the force field (computed every 10 ps of simulation). **(A)** Replica 1; **(B)** replica 2 (S1 meta-stable state, S2 unfavorable state, S3 pseudo-orthosteric state); **(C)** replica 3; **(D)** replica 4; **(E)** replica 5.

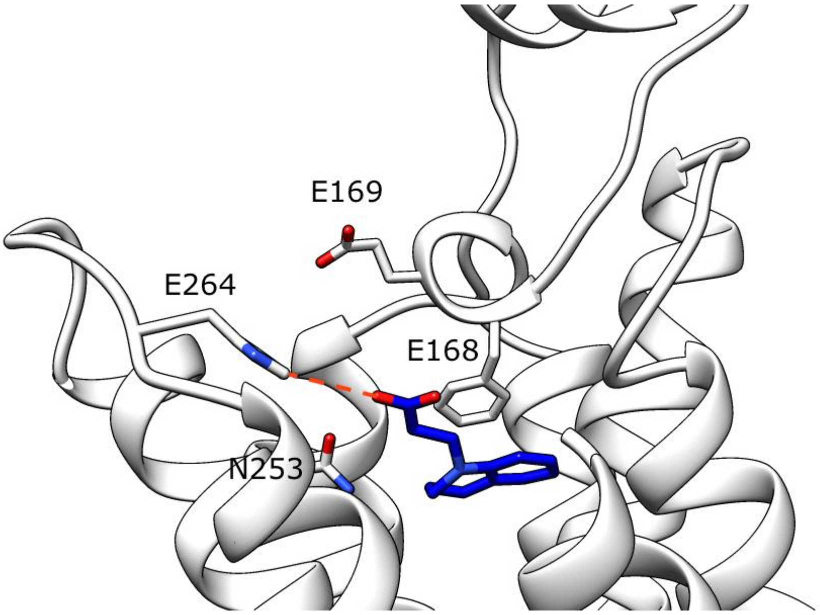

Figure S3. Compound ZB1854 at the end of SuMD replica 5 (on A_{2A} AR apo form). The fragment electrostatically interacts with H264 (EL3) and makes hydrophobic contacts with F168 (EL2).

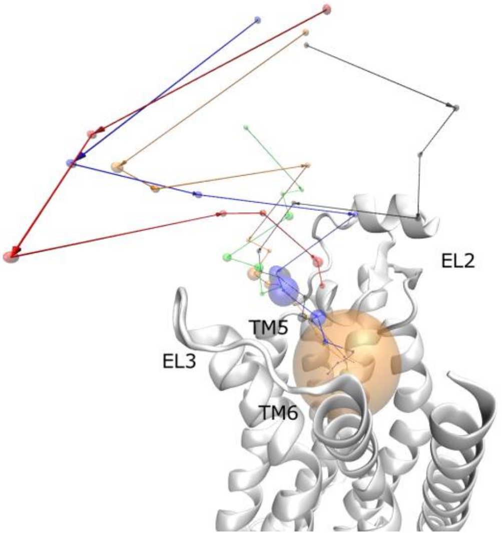

Figure S4. Caffeine - A_{2A} AR recognition pathways obtained from five SuMD simulation replicas. Replica 1 is red color coded; replica 2 is blue color coded; replica 3 is green color coded; replica 4 is orange color coded; replica 5 is black color coded.

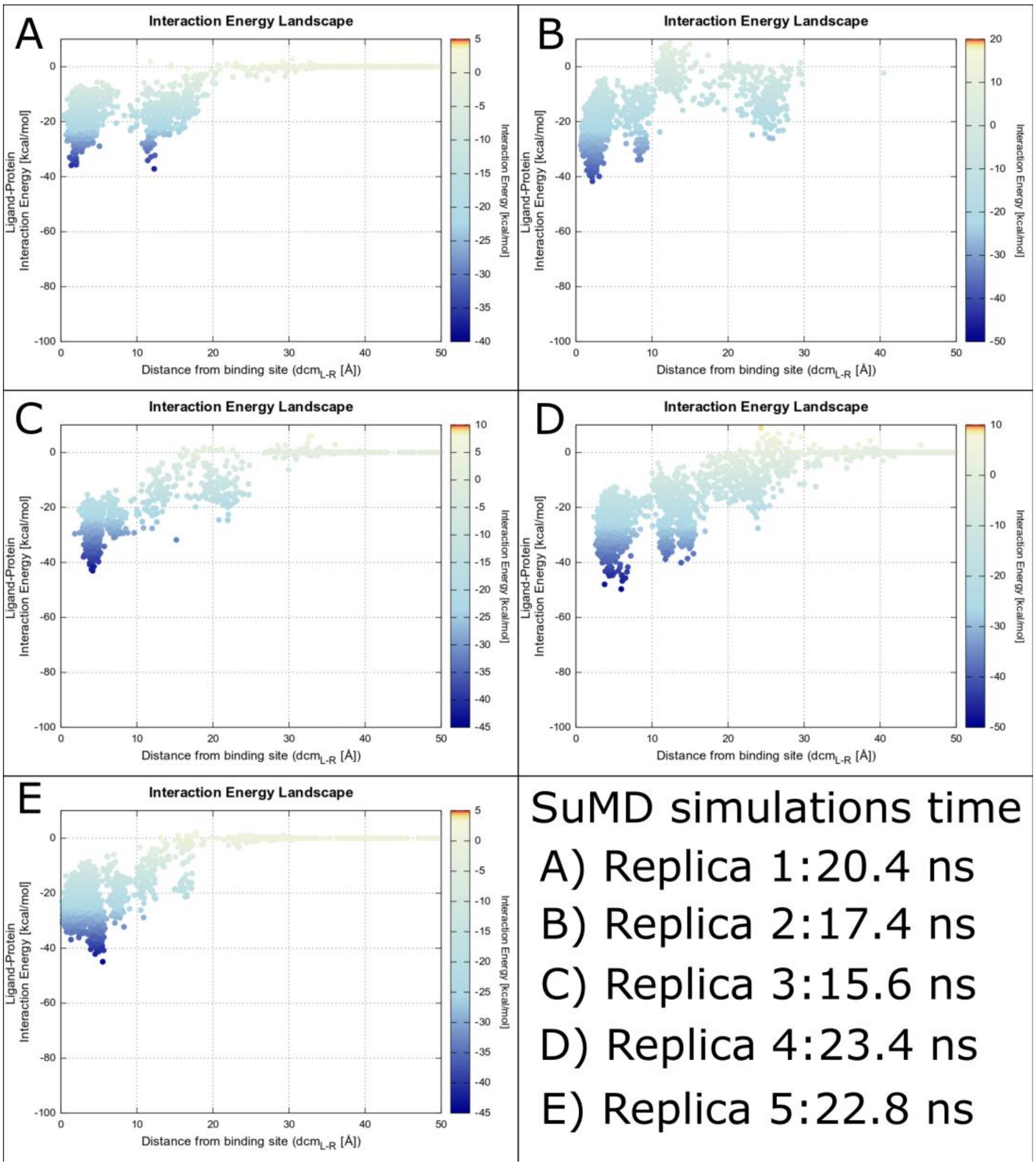

Figure S5. Caffeine - A_{2A} AR (apo form) recognition energy landscapes for the five SuMD replicas performed. On the x axis is reported the ligand distance from the orthosteric site, while the y axis shows the intermolecular interaction extracted from the force field (computed every 10 ps of simulation). **(A)** Replica 1; **(B)** replica 2; **(C)** replica 3; **(D)** replica 4; **(E)** replica 5.

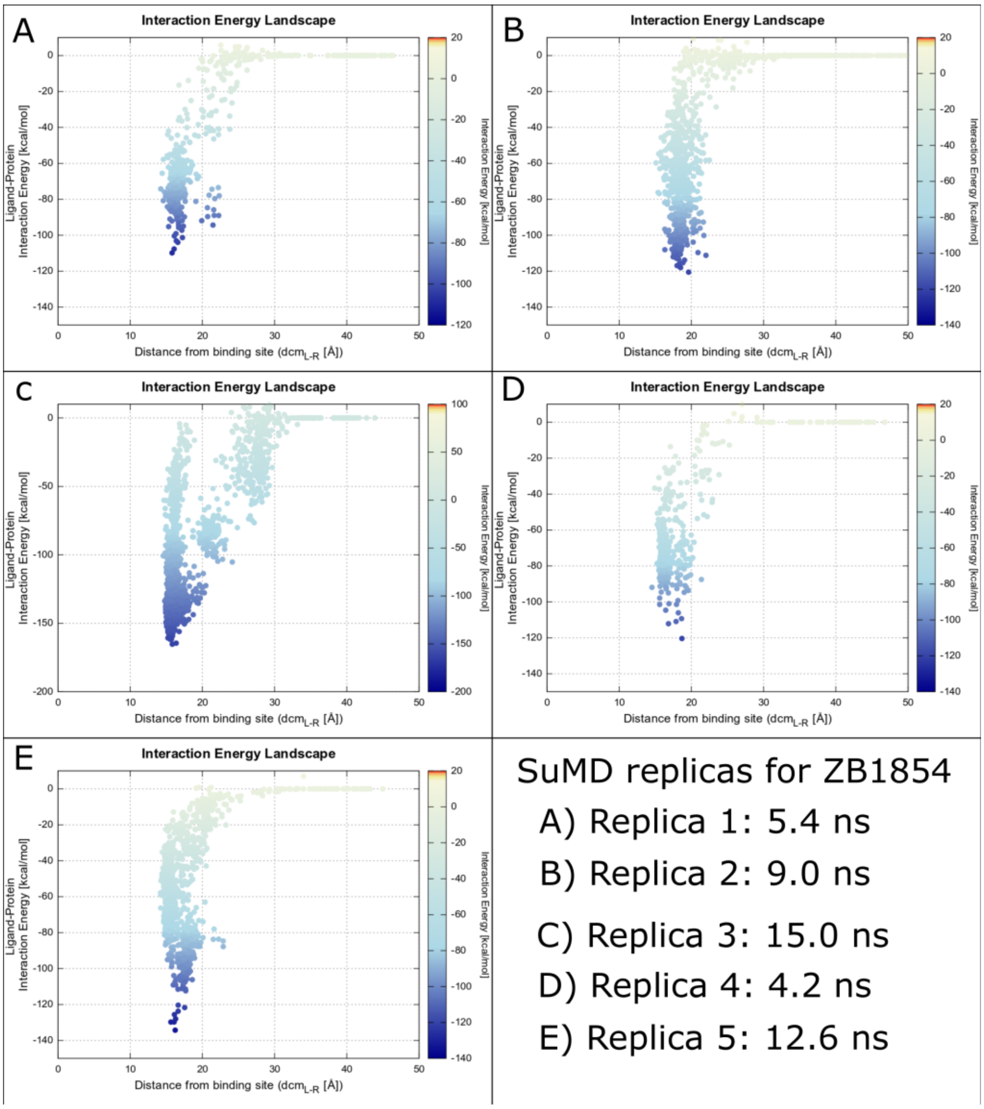

Figure S6. ZB1854 - A_{2A} AR (holo form) recognition energy landscapes for the five SuMD replicas performed. On the x axis is reported the ligand distance from the orthosteric site, while the y axis shows the intermolecular interaction extracted from the force field (computed every 10 ps of simulation). **(A)** Replica 1; **(B)** replica 2; **(C)** replica 3; **(D)** replica 4; **(E)** replica 5.

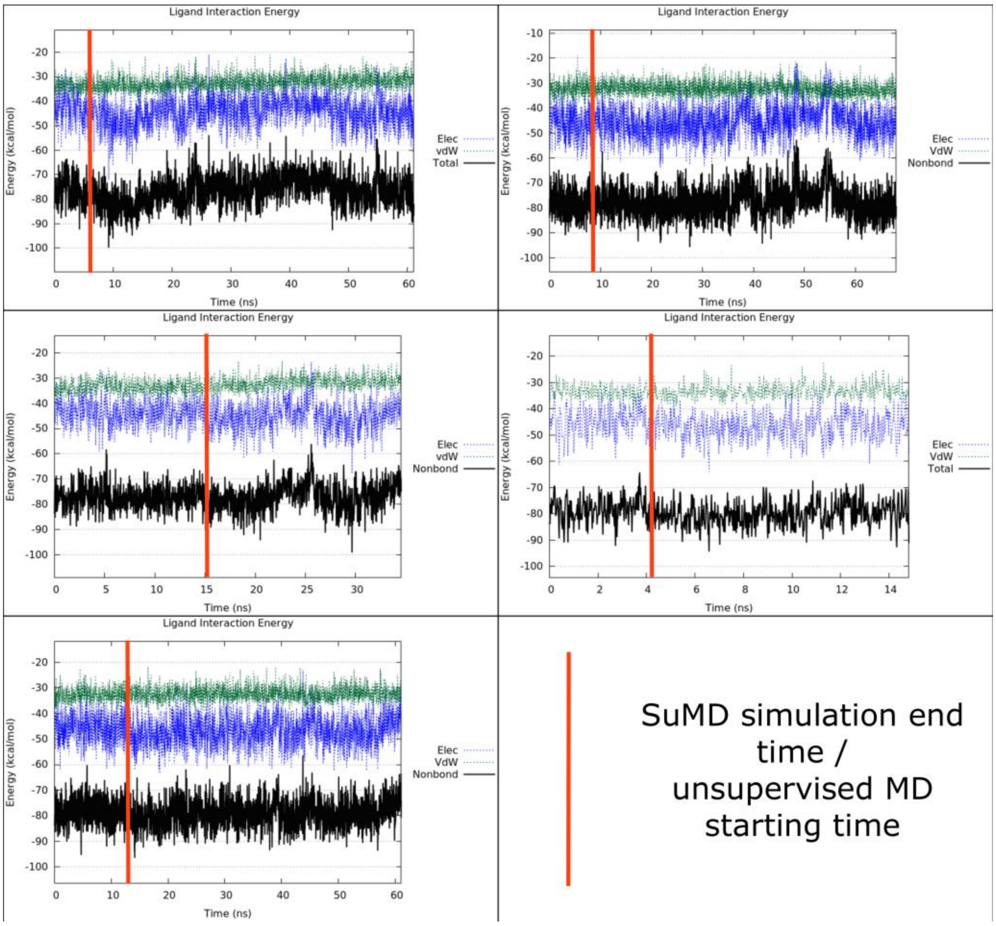

Figure S7. NECA - A_{2A} AR (holo form) orthosteric interactions during SuMD and unsupervised MD simulations of ZB1854. Red lines indicate the end of the supervision algorithm. **(A)** Replica 1; **(B)** replica 2; **(C)** replica 3; **(D)** replica 4; **(E)** replica 5.

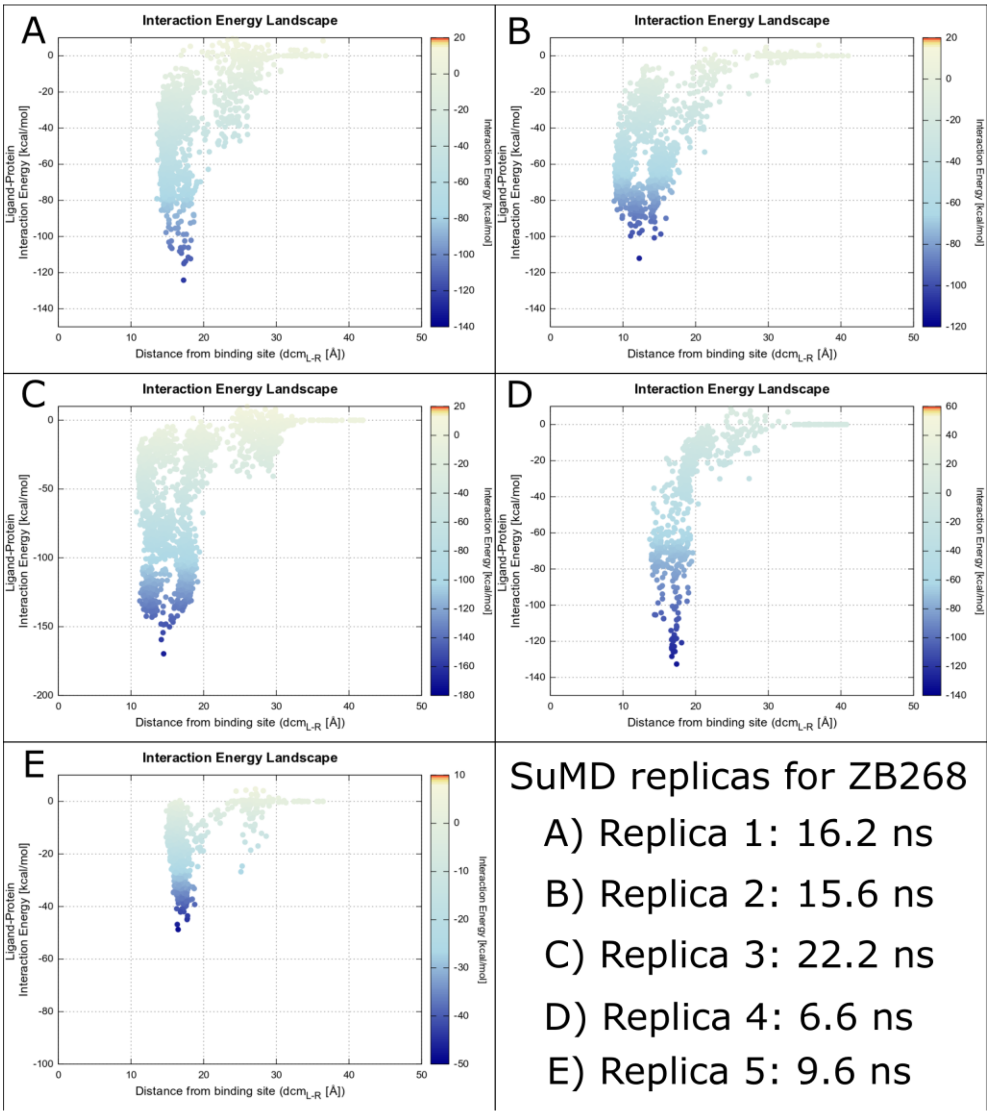

Figure S8. ZB268 - A$_{2A}$ AR recognition energy landscapes for the five SuMD replicas performed. On the x axis is reported the ligand distance from the orthosteric site, while the y axis shows the intermolecular interaction extracted from the force field (computed every 10 ps of simulation). **(A)** Replica 1; **(B)** replica 2; **(C)** replica 3; **(D)** replica 4; **(E)** replica 5.

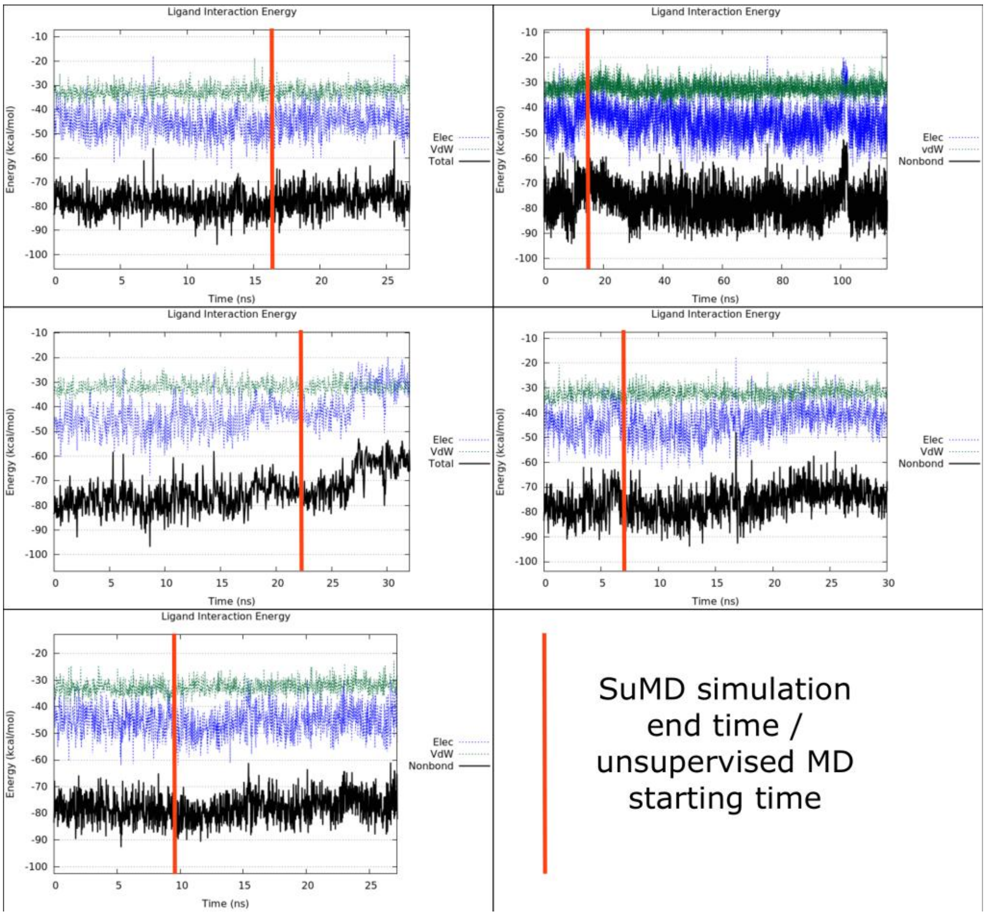

Figure S9. NECA - A2A AR orthosteric interactions during SUMD and unsupervised MD simulations of ZB268. Red lines indicate the end of the supervision algorithm. (A) Replica 1; (B) replica 2; (C) replica 3; (D) replica 4; (E) replica 5.

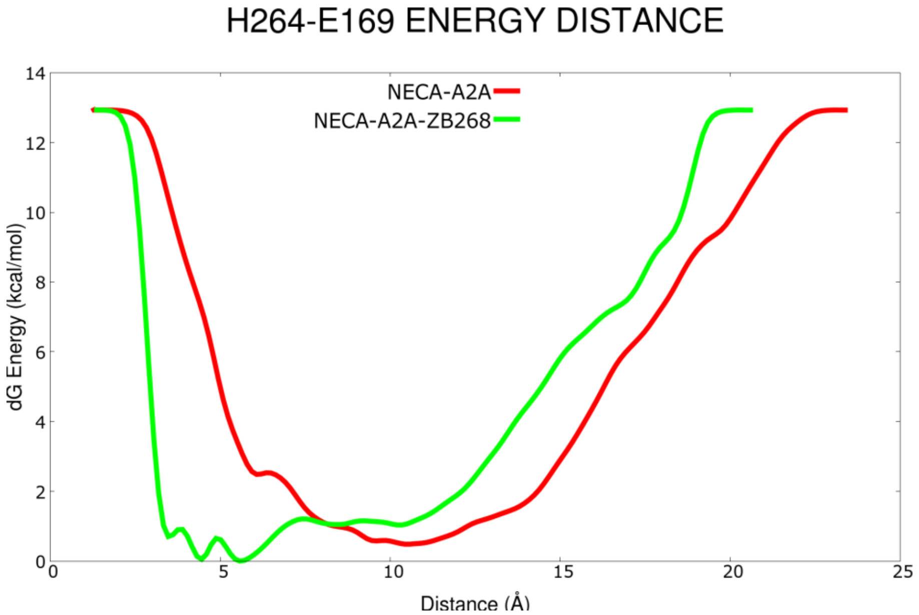

Figure S10. Stability of the salt bridge between H264 and E169 according to metadynamics simulations (see Methods section for details). Red line: A2A AR holo form (complex with NECA); green line: A2A AR holo form with NECA + ZB268 from SuMD replica 5.
